# Plerixafor as a preemptive or salvage therapy for healthy donors with poor mobilization of hematopoietic stem cells

**DOI:** 10.1038/s41409-022-01789-1

**Published:** 2022-09-08

**Authors:** Pu Kuang, Ting Lin, Xinchuan Chen, Yunfan Yang, Jie Ji, Tian Dong, Jie Wang, Yan Li, Ting Niu

**Affiliations:** Department of Hematology, Institute of Hematology, West China Hospital of Sichuan University, Chengdu, PR China

**Keywords:** Haematopoietic stem cells, Stem-cell research

## To the Editor:

The CD34+ cell number is the most important factor for successful engraftment after either autologous or allogeneic hematopoietic stem cell transplantation (HSCT) [1, 2]. The minimal CD34+ cell number needed for HSCT is widely accepted to be 2.0 × 10^6^/kg [3]. Poor mobilization is related to delayed engraftment, prolonged hospitalization, infection or even early death [1]. Unlike conventional strategies, such as priming chemotherapy and avoiding myelotoxic agent exposure, plerixafor effectively increases the hematopoietic stem cell (HSC) yield by an average of 2.7-fold by selectively competing with SDF-1, a major molecule for HSCs homing at CXC chemokine receptor 4 (CXCR-4) expressed on stromal cells in the hematopoietic microenvironment [4]. Patients who failed previous mobilization were able to achieve the minimum requirement for HSCT after salvage treatment with plerixafor. Plerixafor also improved the mobilization efficiency in patients at high risk of mobilization failure. According to the ASBMT guidelines, plerixafor is recommended for preemptive purposes for low CD34+ numbers (10/μl) in peripheral blood before harvest [3].

Poor mobilization was also seen in 2–5% of healthy donors and required further intervention [5]. Salvage methods include increasing the circulating volume and harvest session. All these methods can put more stress on human resource allocation and result in more damage to healthy donors [6]. Plerixafor, as an effective and safe method to escalate HSC mobilization efficiency, provides a potential for preemptive intervention for healthy donors at risk of mobilization failure [7]. Herein, we report our experience and summarize the safety profile of plerixafor used in this scenario.

We retrospectively reviewed our records for healthy donors from January 2018 to December 2021. According to our protocol, healthy donors who used plerixafor in addition to G-CSF before harvest should agree with one of the following criteria: 1. Prior history of poor mobilization (defined as <2 × 10^6^/kg CD34+ mononuclear cells (MNCs) on day 1 of harvest); 2. Prior engraftment failure; 3. Age over 60 years; 4. The CD34+ cell count in peripheral blood one day before harvest was <15/µl. All recipients and donors provided written informed consent in accordance with the Helsinki protocol. Donors were mobilized with 7.5–10 μg/kg G-CSF for five consecutive days. The CD34+ cell count in peripheral blood of donors was detected by BD FACS Canto II flow cytometer on day 4. For preemptive use, plerixafor was administered once subcutaneously on day 4, usually 11 h before harvest, at a median dose of 24 (12–24) mg. For salvage use, plerixafor was used at night on day 5. HSCs were collected by COM. TEC Blood Cell Separator (Fresenius Kabi, Germany) on day 5 and/or 6. Sodium citrate anticoagulant was used as an anticoagulant. Calcium gluconate was routinely infused into the donors during the collection process to prevent hypocalcemia.

After conditioning regimen, the grafts were infused on the same day as HSC collection. The target CD34+ cell number was 2 × 10^6^/kg. For haploidentical donor (HID) transplantation, we tried to attain a higher CD34+ cell number of 4 × 10^6^/kg. Neutrophil engraftment was defined as a neutrophil count over 500/µl for 3 days. Platelet engraftment was defined as the first day of platelet count over 20 × 10^9^/L without platelet transfusion for 7 days. For HLA-matched transplantation, cyclosporine A and methotrexate were used to prevent acute graft versus host disease (aGVHD). For HID transplantation, GVHD prophylaxis includes post-transplantation high-dose cyclophosphamide, cyclosporine continuously infused from day +5 and shifted to oral dosage whenever possible, and mycophenolate mofetil (MMF) given for 4–6 weeks starting on day +5.

Adverse events of donors were classified and recorded according to the Common Terminology Criteria of Adverse Events (CTCAE, Version 5.0). Acute graft versus host diseases were recorded and graded according to MAGIC criteria [8]. The cumulative incidences of engraftment were calculated with the Kaplan–Meier method. The cumulative incidence of acute GVHD was estimated with Gray’s test using a competing risk model with all-cause mortality considered a competing event.

Eighteen healthy donors were included in this study. One was salvage use, and 17 were preemptive use. Their median age is 50 (27–71) years and median body weight is 56 (40–90) kg. Peripheral blood CD34+ cell counts were obtained from 18 donors. The median CD34+ cell count was 8.66 (5.19–14.43)/μl. For donors using plerixafor for preemptive purposes, all donors reached the required number for HSCT in one harvest session. For one donor who received plerixafor as salvage therapy, two harvest sessions were performed. The median MNC and CD34+ MNC counts of the harvest product were 312240 (166919–465511)/µl and 1598.5 (1038–3892)/µl, respectively. The median number of CD34+ MNCs was 6.10 × 10^6^/kg (see Table 1). One recipient died from a gram-negative bacterial bloodstream infection that was resistant to meropenem 10 days after transplantation and therefore was not included for engraftment analysis. The cumulative incidences of neutrophil and platelet engraftment at 30 days were 94.1% and 86.3%, respectively. The median times of neutrophil and platelet engraftment were 13 and 15 days, respectively (Supplementary Fig. 1A). One graft failure occurred, and the recipient died from sepsis 45 days after transplantation.

**Table 1:** Baseline characteristic of transplantation recipients.

	Level	Number (%)
Number of recipients		18
Gender	Male	5 (27.8)
	Female	13 (72.2)
Age (median[IQR])		38.50 [27.00, 50.75]
Underlying disease	ALL	4 (22.2)
	AML	6 (33.3)
	CML	1 (5.6)
	MDS	3 (16.7)
	MPAL	1 (5.6)
	PMF	1 (5.6)
	SAA	2 (11.1)
Disease status	CR>1	1 (5.6)
	CR1	12 (66.7)
	De novo	4 (22.2)
	RR	1 (5.6)
Donor type	HID	9 (50.0)
	MRD	9 (50.0)
Relation to donor	Parent	1 (5.6)
	Child	7 (38.9)
	Sibling	10 (55.6)
HLA match status	5/10	9 (50.0)
	6/10	5 (27.8)
	6/12	1 (5.6)
	8/12	1 (5.6)
	9/12	1 (5.6)
	10/10	1 (5.6)
Conditioning regimen type	MAC	16 (88.9)
	NMA	2 (11.1)
Conditioning regimen protocol	BFA	15 (83.3)
	BFA + TT	1 (5.6)
	FluCyATG	2 (11.1)
CD34 + cell counts in PB (median[IQR])		8.66 [7.22, 12.30]
Purpose for plerixafor use	Preemptive	17 (94.4)
	Salvage	1 (5.6)
TNC (x10^8^/kg) (median[IQR])		11.77 [9.93, 14.24]
CD34 (x10^6^/kg) (median[IQR])		6.10 [4.73, 7.58]

*ALL* acute lymphoblastic leukemia, *AML* acute myeloid leukemia, *BFA* busulfan 3.2 mg/kg × 4, fludarabine 30 mg/m^2^ × 5, cytarabine 1000–1500 mg/m^2^, *CML* chronic myelogenous leukemia, *FluCyATG* fludarabine 30 mg/m^2^ × 4, cyclophosphamide 300 mg/m^2^ × 4, *ATG* antithymoglobulin 2.5 mg/kg × 4, *IQR* inter quartile range, *MAC* myeloablative conditioning, *MDS* myelodysplasia syndrome, *MPAL* mixed phenotype acute leukemia, *NMA* nonmyeloablative conditioning, *PMF* primary myelofibrosis, *SAA* severe aplastic anemia, *TT* thiotepa 30 mg/kg × 2.

Five patients developed grade 2–4 aGVHD, and four patients developed grade 3–4 aGVHD. The estimated incidences of grade 2–4 and grade 3–4 aGVHD were 27.8% and 22.2%, respectively (see Supplementary Fig. 1B). Two patients who developed severe aGVHD died from transplantation-related microangiopathy and lung infection on days 101 and 128 after transplantation, respectively. The most common adverse reactions of any grade for donors were diarrhea (22.2%) and abdominal pain (16.7%), and no adverse reactions of grade >3 were observed.

Plerixafor appeared to be safe and well tolerated in healthy donors. The engraftment did not appear to be impaired with plerixafor-mobilized grafts, and the incidence of aGVHD was also similar to our historical cohort. For preemptive purpose, all donors in this setting only need one apheresis session to reach the required number of CD34+ cells, which apparently saves human resources and avoids overtime working. For salvage purposes, by using plerixafor, excessive staff allocation for marrow collection, risks of anesthesia and chronic pain on puncture sites can be avoided [9].

Although a minimum of 2.0 × 10^6^/kg CD34+ cells is required for successful transplantation, a higher dose, such as 4.0 or 5.0 × 10^6^/kg, is recommended for faster engraftment and less graft failure [10]. Moreover, for HID transplantation, more CD34+ cells are usually required better engraftment. In our study, all recipients from these donors finally reached the minimal number of 4 × 10^6^/kg.

The most widely accepted dose of plerixafor is 0.24 mg/kg and capped at 24 mg. However, a higher dose, such as 0.48 mg/kg, has also been tested in healthy volunteers, showing limited dose-related toxicity but only a moderate increase in CD34+ cell numbers in peripheral blood [11]. In our study, most healthy donors received a plerixafor dose higher than 0.24 mg/kg. We did not observe any correlation between the dose of plerixafor and the CD34+ yield.

According to the 2019 EBMT guidelines, for autologous HSC mobilization, the threshold of CD34+ cell count for preemptive intervention with plerixafor is 10/μL. When the CD34+ cell count is 10–20/μL, the decision of plerixafor use should be based on the patient’s history and clinician’s judgment [12]. In our study, when the peripheral blood CD34+ cell count was <15/μL, the preemptive intervention of plerixafor was initiated. The threshold value we set was arbitrary. Apparently, the single factor was not enough to discriminate donors with poor mobilization risk. We hope that more research in the future can propose a more precise model, which includes the CD34+ cell count, donor age, and recipient body weight, to predict the CD34+ MNC yield so that the initiation of plerixafor can be more specific.

To our knowledge, this article summarized a relatively large, although not the largest, number of healthy donors who mobilized with plerixafor and G-CSF for preemptive or salvage purposes. We successfully avoided mobilization failure by preemptively using plerixafor in healthy donors at high risk.

## Supplementary information

Supplement figure

## Data Availability

The datasets generated during the current study are available from the corresponding author.
