# Supplementary material for: Plerixafor as a preemptive or salvage therapy for healthy donors with poor mobilization of hematopoietic stem cells
Source: Bone Marrow Transplant. 2022 Sep 8;57(11):1737–9. doi: 10.1038/s41409-022-01789-1 (PMC9630128; doi:10.1038/s41409-022-01789-1)
Supplement: Supplementary file 1 — Supplement figure [file 41409_2022_1789_MOESM1_ESM.docx]

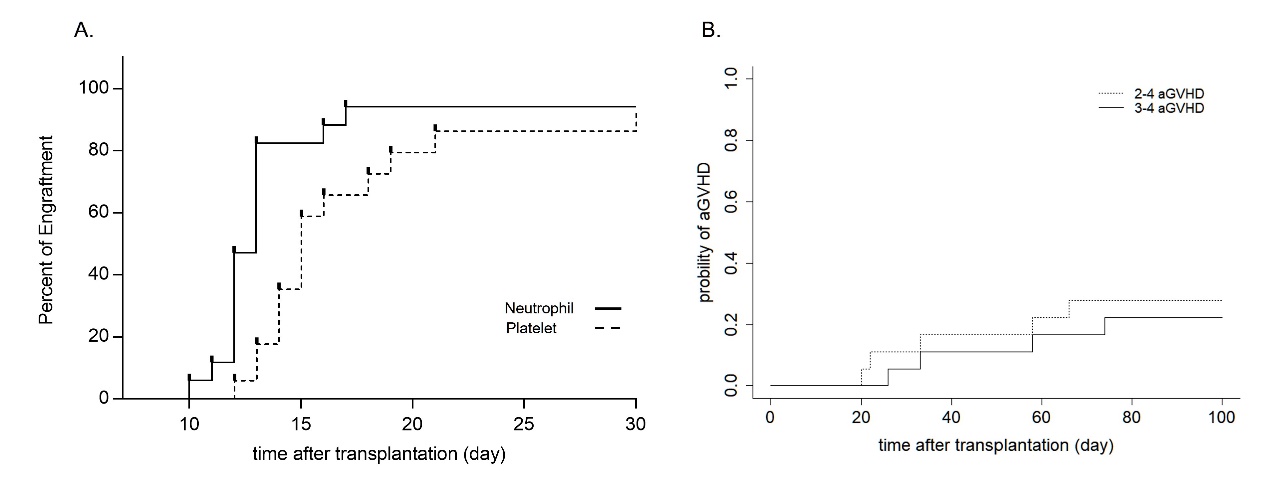


**Supplementary Figure 1A:** **Cumulative incidence of neutrophil and platelet engraftment.**

**Supplementary Figure 1B: The incidence of acute graft versus host disease (aGVHD) in recipients.**
